# Accuracy of four models and update strategies to estimate liver tumor motion from external respiratory motion

**DOI:** 10.3389/fonc.2024.1470650

**Published:** 2024-09-24

**Authors:** Payam Samadi Miandoab, Esben Worm, Rune Hansen, Britta Weber, Morten Høyer, Shahyar Saramad, Saeed Setayeshi, Per Rugaard Poulsen

**Affiliations:** ^1^ Department of Oncology, Aarhus University Hospital, Aarhus, Denmark; ^2^ Department of Energy Engineering and Physics, Amirkabir University of Technology, Tehran, Iran; ^3^ Danish Centre for Particle Therapy, Aarhus University Hospital, Aarhus, Denmark

**Keywords:** tumor motion monitoring, intrafraction motion, external-internal motion correlation, real-time tumor tracking, liver radiotherapy

## Abstract

**Background:**

This study investigates different strategies for estimating internal liver tumor motion during radiotherapy based on continuous monitoring of external respiratory motion combined with sparse internal imaging.

**Methods:**

Fifteen patients underwent three-fraction stereotactic liver radiotherapy. The 3D internal tumor motion (INT) was monitored by electromagnetic transponders while a camera monitored the external marker block motion (EXT). The ability of four external-internal correlation models (ECM) to estimate INT as function of EXT was investigated: a simple linear model (ECM1), an augmented linear model (ECM2), an augmented quadratic model (ECM3), and an extended quadratic model (ECM4). Each ECM was constructed by fitting INT and EXT during the first 60s of each fraction. The fit accuracy was calculated as the root-mean-square error (RMSE) between ECM-estimated and actual tumor motion. Next, the RMSE of the ECM-estimated tumor motion throughout the fractions was calculated for four simulated ECM update strategies: (A) no update, 0.33Hz internal sampling with continuous update of either (B) all ECM parameters based on the last 2 minutes samples or (C) only the baseline term based on the last 5 samples, (D) full ECM update every minute using 20s continuous internal sampling.

**Results:**

The augmented quadratic ECM3 had best fit accuracy with mean (± SD)) RMSEs of 0.32 ± 0.11mm (left-right, LR), 0.79 ± 0.30mm (cranio-caudal, CC) and 0.56 ± 0.31mm (anterior-posterior, AP). However, the simpler augmented linear ECM2 combined with frequent baseline updates (update strategy C) gave best motion estimations with mean RMSEs of 0.41 ± 0.14mm (LR), 1.02 ± 0.33mm (CC) and 0.78 ± 0.48mm (AP). This was significantly better than all other ECM-update strategy combinations for CC motion (Wilcoxon signed rank p<0.05).

**Conclusion:**

The augmented linear ECM2 combined with frequent baseline updates provided the best compromise between fit accuracy and robustness towards irregular motion. It allows accurate internal motion monitoring by combining external motioning with sparse 0.33Hz kV imaging, which is available at conventional linacs.

## 1 Introduction

Radiotherapy is a cornerstone of cancer treatment that relies on the ability to accurately irradiate a tumor volume while minimizing dose to surrounding healthy tissue (1). The treatment accuracy in the abdomen and thorax is challenged by respiratory motion (2–4). Here, real-time adaptive techniques such as respiratory gating or tumor tracking may be utilized to mitigate motion-induced treatment uncertainties (1, 5, 6) but these techniques require real-time tumor motion monitoring. The motion monitoring can rely on either direct internal motion monitoring, external motion monitoring or hybrid methods that combine external respiratory motion monitoring with temporally sparse internal monitoring (1, 5). Techniques for internal monitoring include x-ray fluoroscopy of implanted fiducial markers (7–9), implanted electromagnetic transponders (10–12), and MRI based soft-tissue monitoring (13, 14). External monitoring is typically based on optical monitoring of a marker block placed on the patient’s abdomen (15), a belt with pressure sensor (16), spirometry (17) or surface scanning (18). While direct internal motion monitoring is the most accurate, external monitoring is more widely available at standard equipped conventional linear accelerators where it may be used to guide real-time motion-adaptive techniques such as respiratory gating (1, 19).

Hybrid methods combining internal and external motion monitoring are presently used for tumor tracking in specialized commercial systems such as the Cyberknife Synchrony (20), Vero (21, 22) and Radixact (23, 24). A research hybrid method has also been proposed for a conventionally equipped linear accelerator where sparse 0.33 Hz x-ray imaging was used for internal monitoring during treatment (25). Hybrid methods rely on an external-internal motion correlation model (ECM) that estimates internal motion from measured external motion. ECM construction is typically based on a short period of simultaneous external-internal motion monitoring at the beginning of a treatment fraction, followed by different strategies for ECM validation and update by sparse internal monitoring during the fraction, for example every 1-5 s (Vero) (22), every 3 s (standard Varian TrueBeam accelerator 0.33 Hz imaging), or every 30-60 s (Cyberknife) (1, 21).

Several studies investigated different strategies for hybrid motion monitoring at specialized radiotherapy systems and generally confirmed that hybrid monitoring provides higher accuracy than external monitoring alone while it has shorter latency and gives less imaging dose than full x-ray based internal monitoring (1, 19, 25). However, most studies were either based on artificially generated respiratory traces (22), a very limited number of traces (20), or sparse low frequency (< 1 Hz) internal motion data (16, 18, 22). An exception was a study by Seppenwoolde et al. who analyzed longer series of combined and continuous internal (dual x-ray) and external (Anzai laser system) motion and observed a small trend between ECM update frequency and accuracy (26). However, with an average length of 82 s, the time-series in this study were still short compared to the duration of a full treatment fraction, allowing only short time evaluation of the ECM accuracy.

In simulations of a hybrid monitoring method for a conventional linear accelerator, Bertholet et al. used a dataset of continuous internal electromagnetic transponder motion and external optical monitoring acquired during liver stereotactic body radiotherapy (SBRT) treatments (25). Due to intrafraction baseline shifts, frequent ECM updates by 0.33 Hz internal x-ray monitoring markedly improved the ECM accuracy compared to a scenario with no ECM update. However, the study focused on phantom experiments, simulations and clinical application of a single ECM combined with a simple ECM update strategy to account for baseline shifts, while the impact of different ECM models and model update strategies were not investigated.

The present study is based on the same unique and comprehensive dataset of simultaneous continuous internal electromagnetic transponder motion and external marker block motion as Bertholet et al. (25). The motion data were obtained throughout 45 full treatment fractions of SBRT of tumors in the liver. This study investigates the accuracy of a wider range of ECMs in combination with different ECM update strategies with focus on scenarios that could realistically be implemented for hybrid motion monitoring at a conventionally equipped linear accelerator.

## 2 Materials and methods

### 2.1 Patients and motion monitoring

This study includes internal and external motion data from fifteen patients with primary liver tumors (n=4) or liver metastases (n=11), who received three-fraction liver SBRT guided by implanted electromagnetic transponders in a research protocol approved by the Research Ethics Committee of Central Denmark Region (ref no 1-10-72-175-14). The treatments have previously been thoroughly described (12). In short, each treatment was delivered with exhale respiratory gating during free-breathing using a TrueBeam accelerator (Varian Medical Systems, Palo Alto, CA, USA) equipped with electromagnetic monitoring (Calypso, version 3.0, Varian Medical Systems). Three Calypso electromagnetic transponders implanted near the tumor provided real-time tumor (surrogate) 3D motion monitoring throughout each treatment fraction at 25 Hz. After the treatments, Calypso log files that included the centroid transponder motion and couch position in the left-right (LR), cranio-caudal (CC) and anterior-posterior (AP) directions (25 Hz resolution) were synchronized with linear accelerator log files that included the delivered monitor units (MU) and beam-on/off status (50 Hz resolution). It resulted in the centroid transponder motion, couch corrections, and accelerator parameters during the gated treatments (12). Note that couch corrections were sometimes performed during a treatment fraction to compensate for tumor baseline drift. For the present study, these couch corrections were subtracted from the recorded transponder motion to obtain the internal transponder motion as it would have been without couch corrections.

During treatment a video camera (Canon LEGRIA HF R606) mounted at the feet-end of the couch recorded the vertical (AP) motion of a marker block (RPM, Varian) on the patient’s abdomen with a frequency of 20 Hz. This in-house developed system resembled the clinical Varian RPM system for optical monitoring of an external marker block but allowed marker block monitoring despite the presence of the Calypso antenna panel. Several large manual changes of the table height performed before patient positioning and after the treatment were recorded with both the camera and the Calypso system. The simultaneous recording of this motion by the camera and the Calypso system was used for retrospective synchronization of the two systems.

### 2.2 Internal and external motion data

For all 45 fractions, the synchronized internal transponder motion and external marker block motion were truncated to only include the time span from start of the acquisition of a pretreatment setup cone-beam computed tomography (CBCT) scan to the end of the last treatment field. Next, all motion traces were examined to identify and remove sections with unreliable or missing motion data caused by the following reasons:

1. Loss of the Calypso signal during setup CBCT acquisition caused by large lateral couch centering that moved the transponders out of the Calypso measurement zone. When the Calypso signal during CBCT acquisition was unavailable, the 60s of motion data immediately before the Calypso signal loss was added to the trace as replacement as 60s corresponded to the CBCT scan duration.
2. Erratic Calypso signal caused by couch corrections during treatments. Five seconds of motion data before and after each couch correction was removed.
3. Sudden signal loss from the Calypso or external camera systems which occurred in a few occasions. Five seconds of motion data before and after each signal loss was removed.
4. Loss of Calypso signal during couch rotations performed before non-coplanar field delivery.

Removal of the sections with unreliable or missing data (with the time kept running) resulted in clean datasets of synchronized internal and external motion. An example is shown in **Supplementary Figure S1**.

### 2.3 External-internal motion correlation models

ECMs rely on consistent correspondence between external and internal motion to estimate the internal 3D motion from the external motion. In this study, four ECMs were investigated, including a simple linear model (ECM 1, Equation 1), an augmented linear model (ECM 2, Equation 2), an augmented quadratic model (ECM 3, Equation 3), and an extended quadratic model (ECM 4, Equation 4).


(1) $$\hat{\text{INT}}\left(\text{t}\right)=\text{a}+\text{b}*\text{EXT}\left(\text{t}\right)$$

(2) $$\hat{\text{INT}}\left(\text{t}\right)=\text{a}+\text{b}*\text{EXT}\left(\text{t}\right)+\text{c}*\text{EXT}\left(\text{t}-\text{τ}\right)$$

(3) $$\hat{\text{INT}}\left(\text{t}\right)=\text{a}+\text{b}*\text{EXT}\left(\text{t}\right)+\text{c}*\text{EXT}\left(\text{t}-\text{τ}\right)+\text{d}*{\text{EXT}}^{2}\left(\text{t}\right)+\text{e}*{\text{EXT}}^{2}\left(\text{t}-\text{τ}\right)+\text{f}*\text{EXT}\left(\text{t}\right)*\text{EXT}\left(\text{t}-\text{τ}\right)$$

(4) $$\hat{\text{INT}}\left(\text{t}\right)=\text{a}+\text{b}*\text{EXT}\left(\text{t}\right)+\text{c}*{\text{EXT}}^{2}\left(\text{t}\right)+\text{d}*\dot{\text{EXT}}\left(\text{t}\right)+\text{e}*{\dot{\text{EXT}}}^{2}\left(\text{t}\right)$$

Here, $\hat{\text{INT}}\left(\text{t}\right)$ is the estimated internal position along each axis (LR, CC, AP) as function of time t, EXT(t) is the external AP position and τ is a fitted time delay accounting for hysteresis or phase differences between internal and external motion. $\dot{\text{EXT}}\left(\text{t}\right)$ is the first derivative of the external position (i.e. velocity) while a, b, c, d, e and f are fitting coefficients. Separate ECMs were fitted for the internal motion in the LR, CC and AP directions. Least squares fitting was applied for all models.

The simple linear model (ECM 1) assumes that each coordinate of internal motion is affine in line with the external motion with a constant offset. The augmented linear model (ECM 2) suggested by Ruan et al. (27) includes a delayed term that accounts for hysteresis motion from external-internal motion datasets. The augmented quadratic model (ECM 3), which was also suggested by Ruan et al. (27), additionally includes quadratic terms to model non-linear behavior. The extended quadratic model (ECM 4) was used at the Vero system (Mitsubishi Heavy Industries, Japan, and BrainLAB AG, Munich, Germany) (22). By including linear and quadratic position and velocity terms this ECM also allows modelling of hysteresis and non-linear motion.

### 2.4 ECM fit accuracy

For each ECM, the ability to fit internal and external motion was first investigated by fitting each model based on the internal and external motion data during the first 60 s of each fraction. The root-mean-square error (RMSE) between the actual internal motion and the ECM fitted internal motion during these 60 s was used to quantify the fit accuracy. The fit accuracy of the different models was compared using the Wilcoxon signed rank test (p < 0.05 considered significant).

### 2.5 ECM estimation of internal motion

For each ECM, four different strategies for fitting and updating the ECM during the remaining part of the fraction were simulated:

- Strategy A: Fit ECM to 60s motion just before treatment. No further ECM update.
- Strategy B: Fit ECM to 60s motion just before treatment. Sample the tumor position every 3s (0.33 Hz) and update all ECM parameters based on the last 2 minutes sampling.
- Strategy C: Fit ECM to 60s motion just before treatment. Sample the tumor position every 3s (0.33 Hz) and update only the constant ECM term (coefficient a in Equations 1-4) based on the last five samples (15s).
- Strategy D: Record the internal motion continuously (25 Hz) for 20s just before each 1-minute interval. Fit the ECM to the motion in the 20s interval and apply it in the 1-minute interval.

Here, Strategy A simulates a situation, where an ECM is built based on pre-treatment imaging alone (e.g., CBCT or fluoroscopy). Strategy B and C continuously update the ECM with a frequency of 0.33 Hz, e.g. by triggered kV imaging on the Varian TrueBeam platform, with Strategy C providing a faster and simpler update that only accounts for baseline drift between internal and external motion. Strategy D could offer full on-demand ECM generation by continuous imaging (e.g. fluoroscopy) immediately before the delivery of each treatment field during a treatment fraction.

### 2.6 ECM estimation accuracy

For each combination of ECM (ECM 1-4) and ECM update strategy (Strategy A-D), the ECM estimation accuracy was quantified by the RMSE between the ECM estimated internal motion and actual internal motion. The RMSE was determined per fraction and compared using the Wilcoxon signed rank test.

## 3 Results

**Figure 1:**
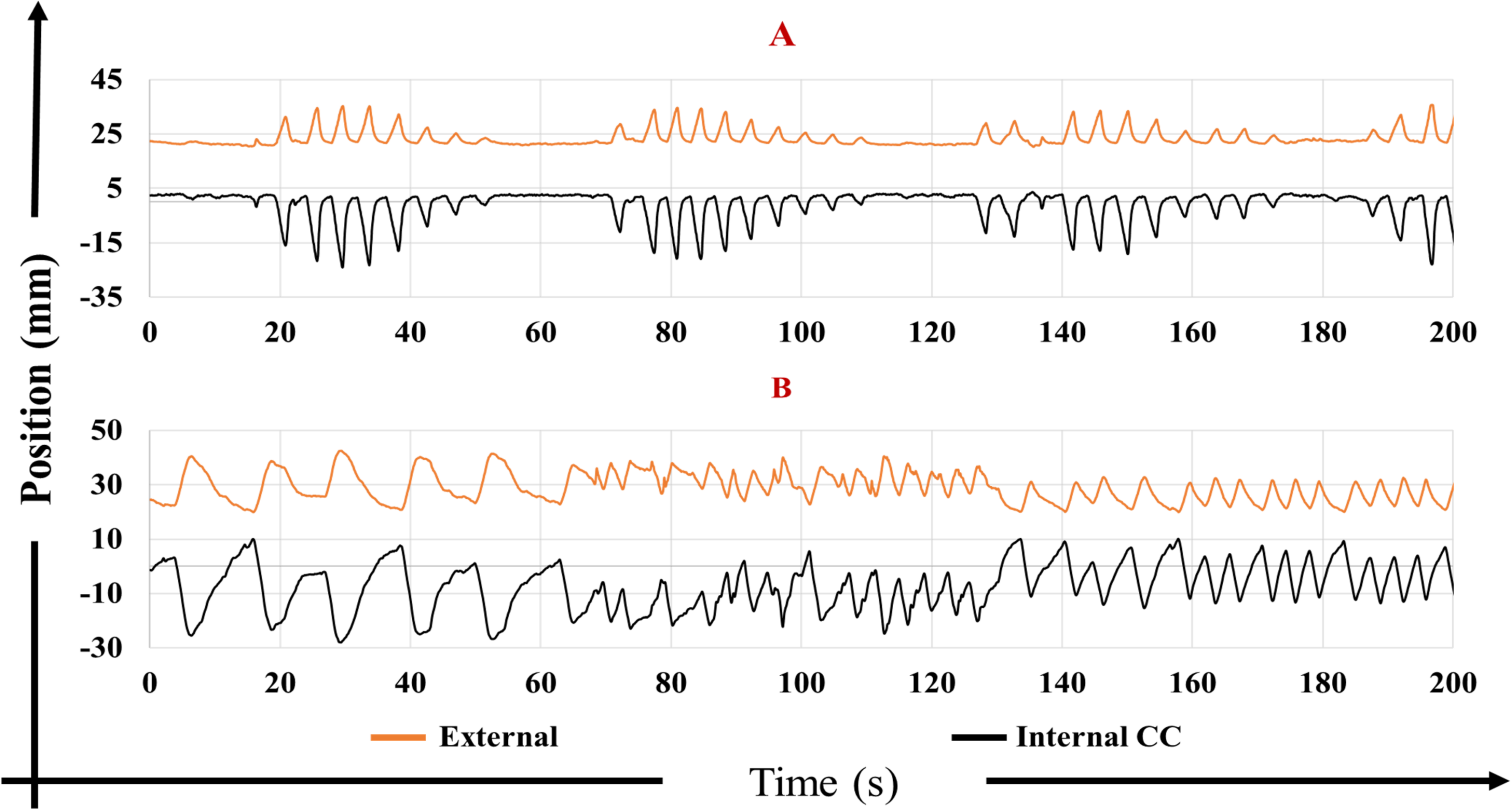
External anterior-posterior marker block motion (orange) and internal cranio-caudal (CC) tumor motion (black) for two motion traces with largely varying **(A)** amplitude and **(B)** frequency.

Three of the 45 fractions were excluded due to corrupted data. The mean (range) duration of the 42 analyzed motion traces was 26 minutes (16-44 minutes) initially and 25 minutes (14-44 minutes) after removal of sections with unreliable or missing data. The cleaned motion traces included missing sections with an average total duration of 4 minutes per motion trace (range: 0-10 minutes). For some motion traces, both the internal and external motion showed large variations in breathing amplitude or frequency as illustrated in **Figure 1**.

### 3.1 ECM fit accuracy

**Figure 2:**
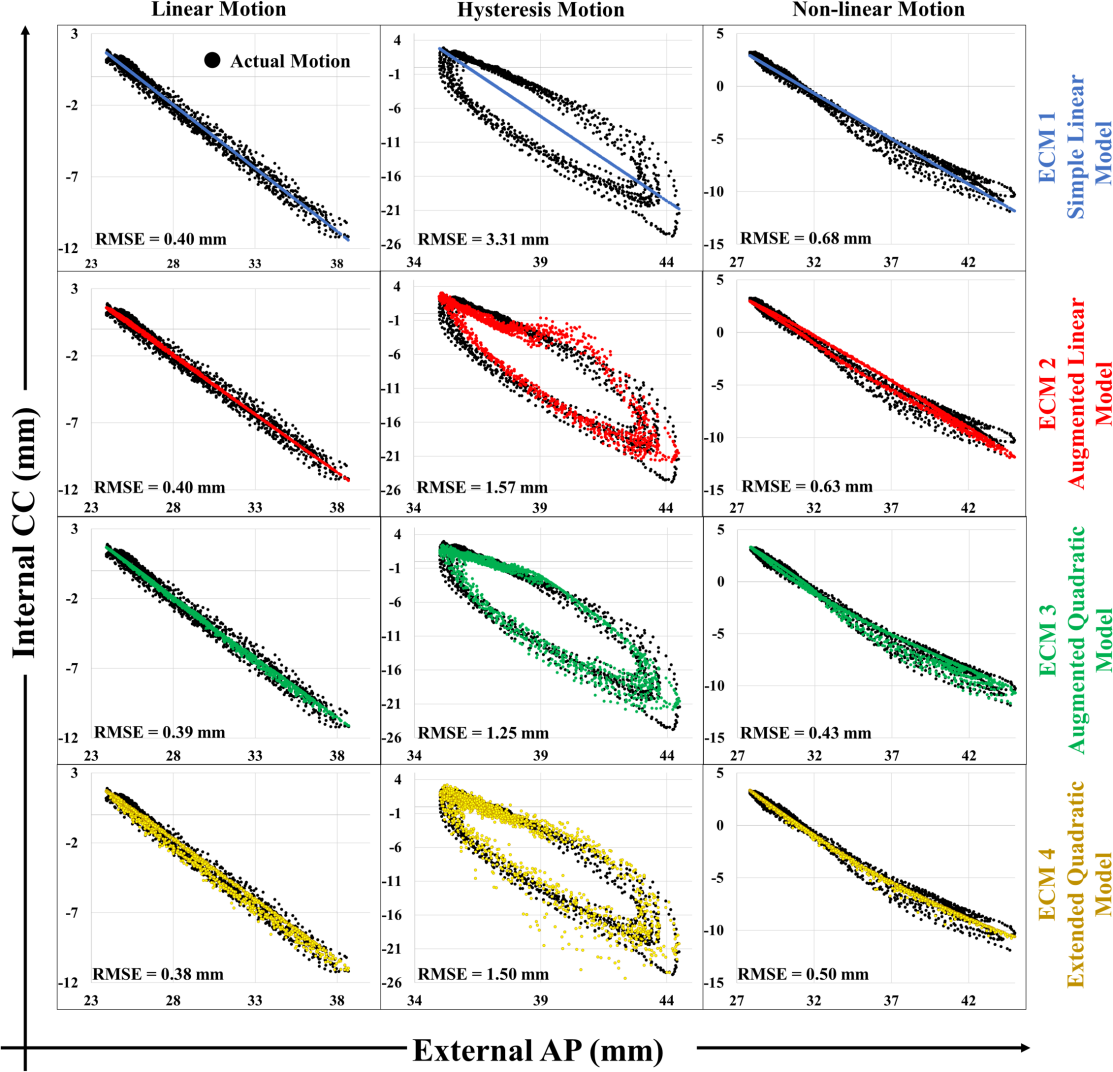
Examples of ECM fits to different types of internal cranio-caudal (CC) motion as function of external anterior-posterior (AP) marker block motion, including linear motion (column 1), hysteresis motion (column 2) and non-linear motion (column 3). Actual motion is shown in black and ECM fits in blue (ECM 1), red (ECM 2), green (ECM 3) and gold (ECM 4). The root-mean-square errors (RMSE) specify the fit accuracy.

**Figure 2** presents characteristic examples of the internal CC tumor motion as function of the external marker block motion during the first minute of three different treatment fractions. The figure also shows the fit to the motion by the four ECMs. For cases with a simple linear correlation between internal and external motion, all models performed equally well (**Figure 2**, column 1). For cases with hysteresis motion, the augmented models (ECM 2 and ECM 3) or the extended model (ECM 4) were needed to estimate the internal motion well (**Figure 2**, column 2). For non-linear motion, quadratic models (ECM 3 and ECM 4) were needed (**Figure 2**, column 3).

**Figure 3:**
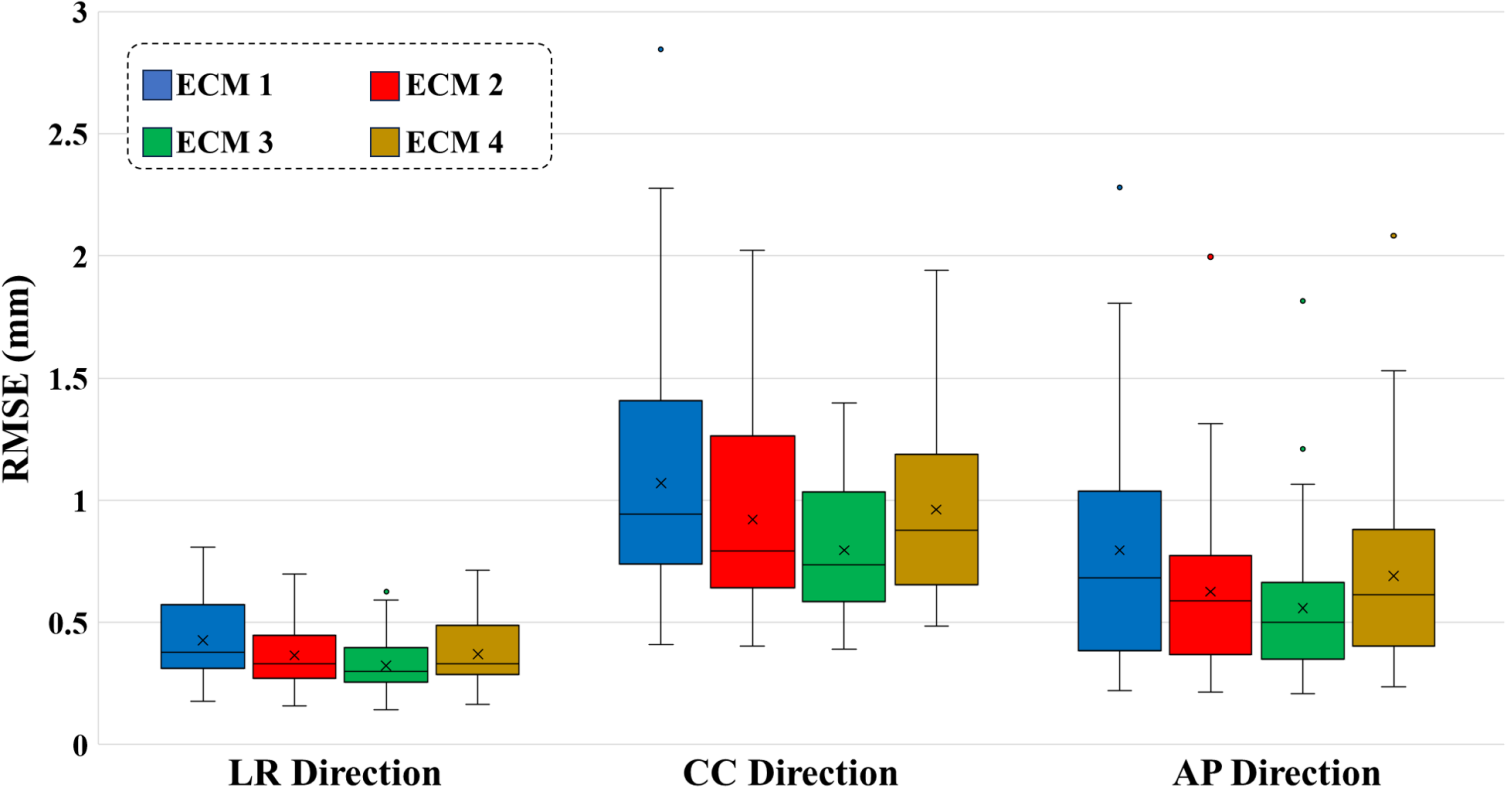
Boxplots of the ECM fit root-mean-square error (RMSE) in the left-right (LR), cranio-caudal (CC), and anterior-posterior (AP) directions during the first 60 s of all treatment fractions. For each box, the horizontal line shows the median, the x-mark shows the mean, bottom and top edges of the box indicate the 25th and 75th percentiles, respectively. Outliers are plotted separately as dots.

**Figure 3** compares the fit accuracy of the 4 ECMs across all fractions. Since ECM 2 is equal to ECM 1 with an additional fitting term, while ECM 3 is equal to ECM 2 with more additional fitting terms, the fit accuracy improved for all traces and all directions when going from ECM 1 to ECM 2 to ECM 3 (**Supplementary Figure S2** shows the CC fit accuracy for all fractions). These improvements were all significant (p < 0.001 along all directions). The fit accuracy of ECM 4 was worse than ECM 3 (p < 0.001 along all directions) and slightly worse than ECM 2 (p = 0.086 (LR), p = 0.002 (CC), p < 0.001 (AP)), see RMSE in **Table 1**. The internal motion was most prominent in the CC direction, where the mean (± standard deviation) fit RMSE across all fractions was 1.07 ± 0.49 mm (ECM 1), 0.92 ± 0.39 mm (ECM 2), 0.79 ± 0.30 mm (ECM 3), and 0.96 ± 0.37 mm (ECM 4) (**Table 1**).

**Table 1:** Mean (± standard deviation) across all treatment fractions of the root-mean-square error (RMSE) for ECM fitting (in first 60s of each fraction) and ECM estimation (in remaining part of the fraction) by all combinations of external-internal motion correlation models (ECM 1-4) with ECM update strategies (A-D).

Directions	Model	ECM fit accuracy (RMSE)	ECM estimation accuracy (RMSE)			
		First 60s	Strategy A	Strategy B	Strategy C	Strategy D
LR (mm)	ECM 1	0.43 ± 0.16	0.84 ± 0.45 **(p < 0.001)**	0.46 ± 0.14 **(p < 0.001)**	0.45 ± 0.18 **(p = 0.002)**	0.49 ± 0.17 **(p < 0.001)**
	**ECM 2**	0.37 ± 0.13	0.83 ± 0.52 **(p < 0.001)**	0.43 ± 0.13 (p = 0.086)	**0.41 ± 0.14**	0.47 ± 0.16 **(p = 0.003)**
	ECM 3	0.32 ± 0.11	0.87 ± 0.52 **(p < 0.001)**	0.44 ± 0.18 **(p = 0.043)**	0.46 ± 0.21 **(p = 0.005)**	0.66 ± 0.83 (**p < 0.001)**
	ECM 4	0.37 ± 0.13	0.84 ± 0.45 **(p < 0.001)**	0.47 ± 0.20 **(p = 0.001)**	0.45 ± 0.19 (p = 0.070)	0.50 ± 0.25 **(p < 0.001)**
CC (mm)	ECM 1	1.07 ± 0.49	2.92 ± 2.43 **(p < 0.001)**	1.21 ± 0.43 **(p < 0.001)**	1.10 ± 0.40 **(p = 0.001)**	1.34 ± 0.51 **(p < 0.001)**
	**ECM 2**	0.92 ± 0.39	2.65 ± 2.19 **(p < 0.001)**	1.09 ± 0.32 **(p = 0.008)**	**1.02 ± 0.33**	1.25 ± 0.46 **(p < 0.001)**
	ECM 3	0.79 ± 0.30	2.74 ± 2.45 **(p < 0.001)**	1.13 ± 0.39 **(p = 0.020)**	1.21 ± 0.60 **(p = 0.029)**	1.70 ± 2.30 **(p < 0.001)**
	ECM 4	0.96 ± 0.37	3.00 ± 2.88 **(p < 0.001)**	1.26 ± 0.54 **(p < 0.001)**	1.17 ± 0.52 **(p < 0.001)**	1.49 ± 0.97 **(p < 0.001)**
AP (mm)	ECM 1	0.80 ± 0.45	1.57 ± 0.79 **(p < 0.001)**	0.91 ± 0.46 **(p < 0.001)**	0.92 ± 0.56 **(p < 0.001)**	0.98 ± 0.57 **(p < 0.001)**
	**ECM 2**	0.63 ± 0.34	1.34 ± 0.61 **(p < 0.001)**	0.77 ± 0.39 (p = 0.67)	**0.78 ± 0.48**	0.88 ± 0.57 **(p = 0.035)**
	ECM 3	0.56 ± 0.31	1.73 ± 1.61 **(p < 0.001)**	0.84 ± 0.61 (p = 0.38)	0.90 ± 0.51 **(p = 0.028)**	1.65 ± 4.22 **(p < 0.001)**
	ECM 4	0.69 ± 0.38	1.67 ± 1.19 **(p < 0.001)**	0.94 ± 0.64 **(p < 0.001)**	0.93 ± 0.51 **(p < 0.001)**	1.20 ± 1.35 **(p < 0.001)**

The p-values compare the RMSE of each ECM-strategy combination with the ECM 2 – Strategy C combination, which in general had lowest RMSE (Wilcoxon signed rank test). Bold type indicates statistically significant differences (p < 0.05).

LR, Left -right; CC, Cranio-caudal; AP, Anterior-posterior.

### 3.2 ECM estimation accuracy

**Figure 4:**
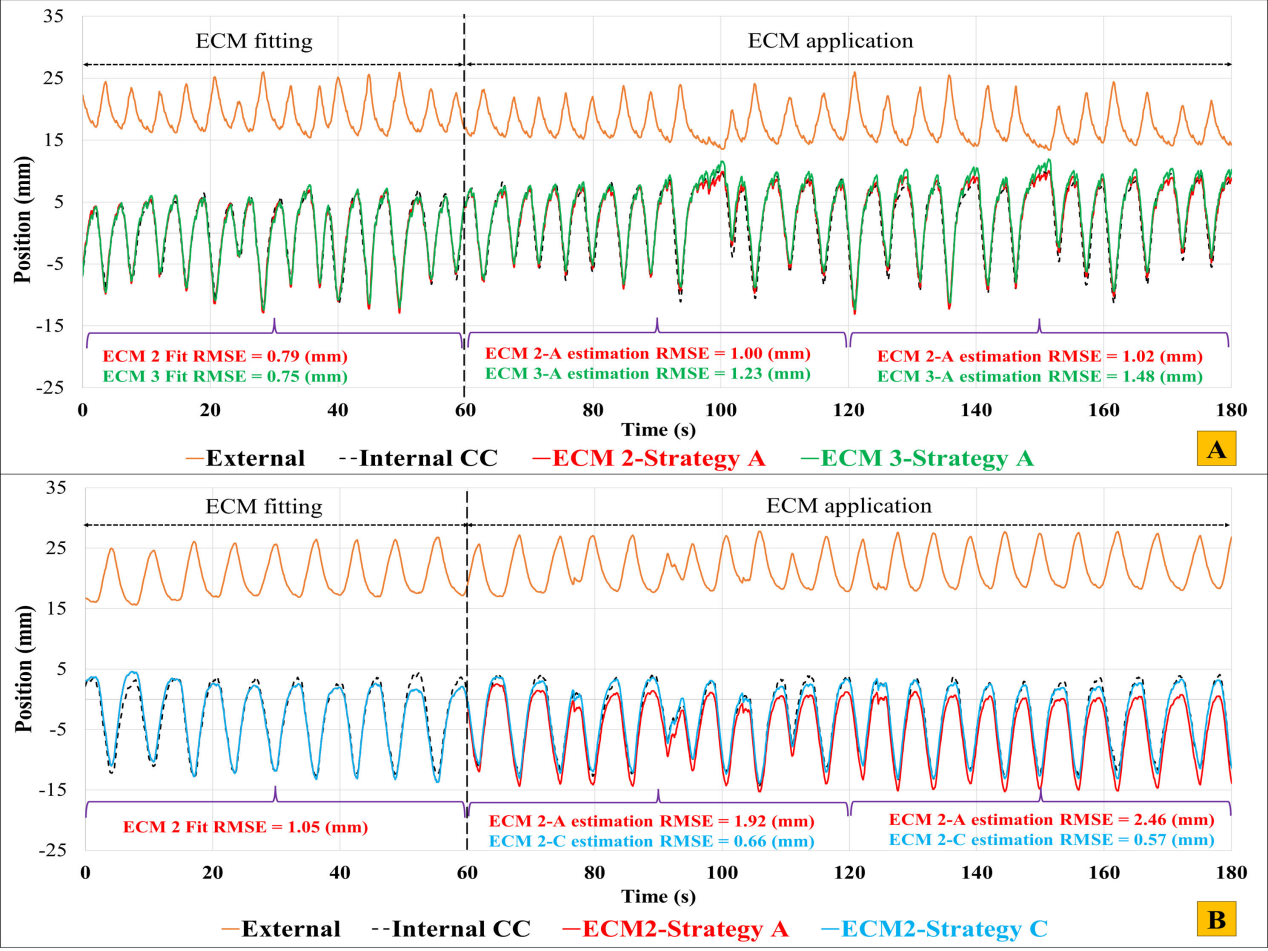
External AP motion (orange) and internal CC motion (black) in the first three minutes of two fractions. **(A)** Result of ECM fit (first 60 s) and subsequent internal motion estimation (60-180 s) using ECM 2 (red) and ECM 3 (green) with no model update (Strategy A). **(B)** Result of using ECM2 with update strategies A (no update, red) and C (baseline update with 0.33 Hz frequency, blue). The root-mean-square errors (RMSE) indicate the fitting accuracy (0-60 s) and ECM estimation accuracy (60-120s and 120-180 s).

**Figure 4** shows two examples of external AP and internal motion in the CC direction in the first three minutes of a fraction along with the estimated internal motion using different ECMs and update strategies. In **Figure 4A**, ECM 2 and ECM3 were fitted to the motion in the first 60s and applied without any updates to estimate the internal motion in the subsequent time period (Strategy A). While the quadratic augmented ECM 3 provided a slightly better fit to the internal motion than the linear augmented ECM 2 for the first 60 s, ECM 2 more accurately estimated the motion in the subsequent period as it tended to be more robust to irregular motion than ECM 3. In **Figure 4B**, ECM 2 was fitted to the motion in the first 60s and then used to estimate internal motion in the subsequent time period by applying update strategies A (no model update) and C (0.33 Hz update of constant ECM term). Here, Strategy C provided considerably better internal motion estimation than Strategy A as the frequent model updates continuously adapted the ECM to baseline drifts between internal and external motion.

**Figure 5:**
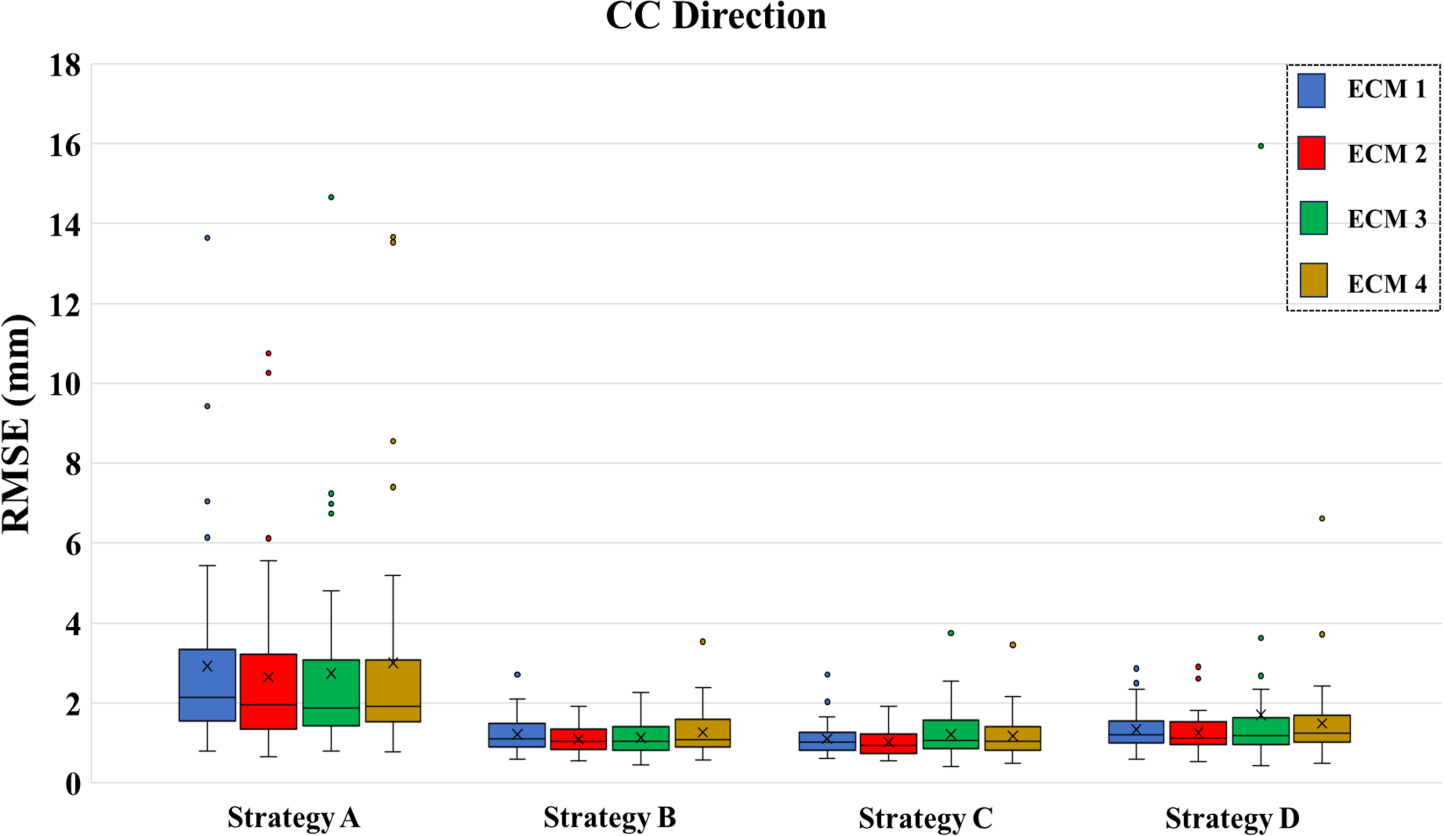
Root-mean-square error (RMSE) of internal motion estimation along the cranio-caudal (CC) direction across all fractions for all combinations of external-internal motion correlation models (ECM1-4) and model update strategies (A–D). For each box, the horizontal line shows the median, the x-mark shows the mean, the bottom and top of the box show the 25^th^ and 75^th^ percentile, respectively. Outliers are shown separately as dots.

**Table 1** shows the mean RMSE across all fractions for all combinations of ECMs (ECM1-4) and model update strategies (A-D). With a mean RMSE of 0.41 ± 0.14 mm (LR), 1.02 ± 0.33 mm (CC) and 0.78 ± 0.48 mm (AP), the augmented linear ECM 2 combined with the frequent baseline update Strategy C provided the best overall estimation of internal motion (highlighted with bold text in **Table 1**). Wilcoxon’s signed rank test showed that this combination was significantly better than all other ECM – strategy combinations except for ECM 2 with Strategy B and ECM 4 with Strategy C in the LR direction and ECMs 2 and 3 with Strategy B in the AP direction (**Table 1**). The results are visually summarized for the CC direction in **Figure 5** that also shows that the frequent update strategies (B and C) combined with the linear augmented ECM 2 were most robust and had no outliers with high RMSE. In contrast, update Strategy A (no model updates) performed far worse than strategies B-D, while the quadratic ECMs 3-4 where less robust towards outliers than the linear ECMs 1-2. **Supplementary Figure S3** shows a part of the fraction with highest RMSE of 16.0 mm (see ECM 3 with Strategy D in **Figure 5**). The high RMSE was due to a large difference between motion during the modelling phases and motion during the estimation phases of Strategy D which especially resulted in poor motion estimation for the quadratic models that tended to amplify irregularities in external and internal motion.

**Figure 6:**
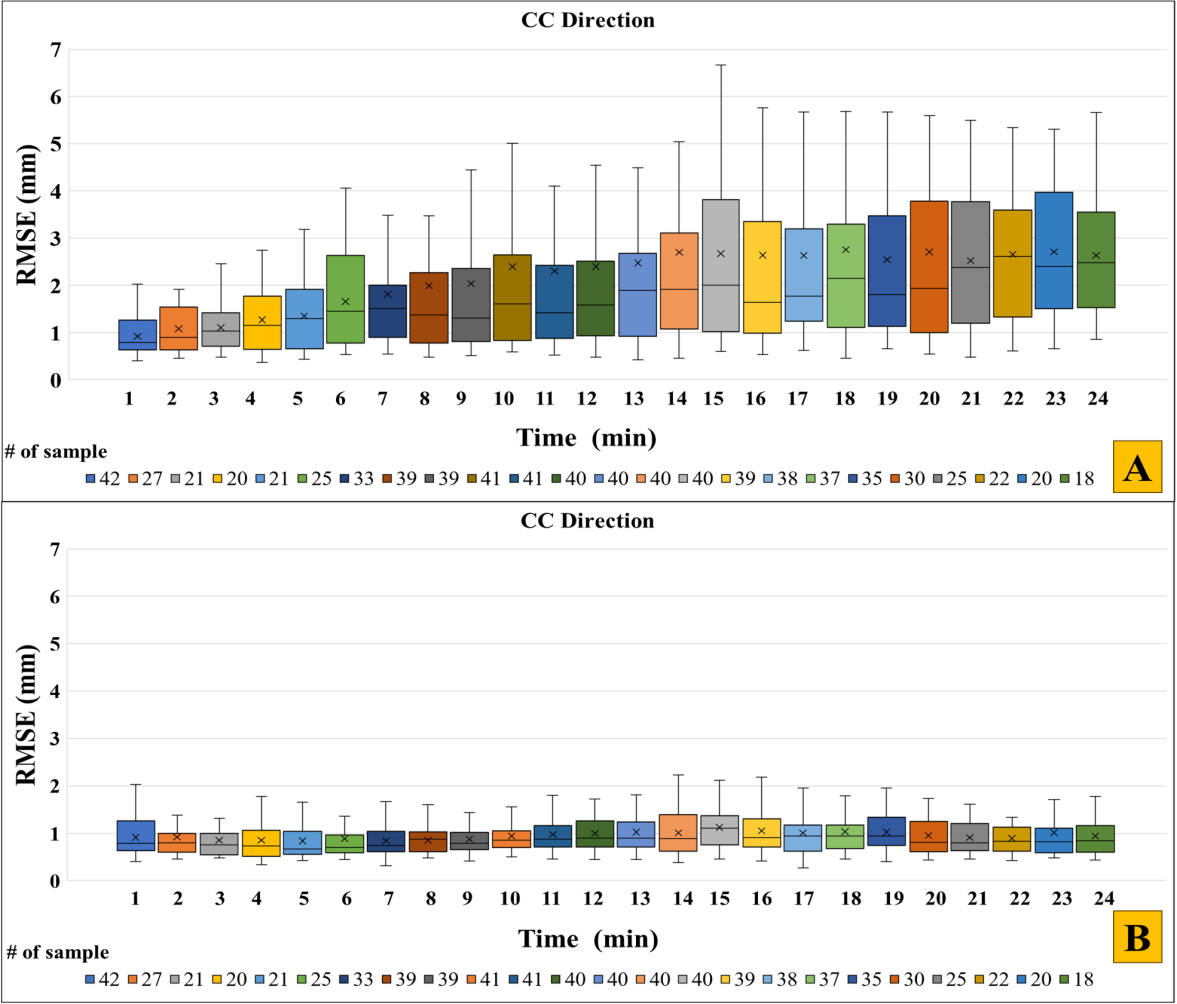
Root-mean-square error (RMSE) across all fractions for each 1-minute interval by the augmented linear model (ECM 2) in the CC-direction for **(A)** update Strategy A (no updates) and **(B)** update Strategy C (baseline update with 0.33 Hz frequency). The first minute shows the ECM fit accuracy of ECM 2, while the remaining minutes indicate the ECM estimation accuracy. For each box, the horizontal line shows the median, the x-mark shows the mean, the bottom and top of the box show the 25^th^ and 75^th^ percentile, respectively. For clarity, outliers are not shown. The number of samples in each boxplot (indicated in the bottom of the figures) varied between the different timepoints due to the sections with unreliable or missing data.

**Figure 6** shows the CC ECM estimation accuracy by the augmented linear ECM 2 combined with update strategies A and C as a function of time across all fractions. Without model updates (**Figure 6A**, Strategy A), the ECM estimation accuracy clearly worsened as the time progressed. With frequent ECM baseline updates (**Figure 6B**, Strategy C), the ECM estimation accuracy stayed on level with the initial fit accuracy even though this strategy only updated the constant term in Equation 2. It shows (1) that baseline drifts were poorly described by the ECM and (2) that except for baseline drifts, an augmented linear ECM constructed before the treatment remained a stable estimator of internal motion during the time span of a typical treatment fraction.

## 4 Discussion

The present study utilized a unique dataset of independently measured internal and external motion throughout 42 liver SBRT fractions to investigate the accuracy of ECM based internal motion estimation. The study included modelling of four different ECMs of varying complexity combined with four different ECM update strategies. All scenarios could realistically be implemented at a conventionally equipped linear accelerator, for example by combining monoscopic kilovoltage intrafraction motion monitoring (KIM) with the external optical signal from the integrated gating system (25). While the more complex augmented quadratic ECM 3 provided the best model fit, the simpler augmented linear ECM 2 with frequent ECM updates (Strategy C) provided the most robust and accurate estimation throughout the fractions.

On a conventionally equipped linear accelerator the gate on/off state in a respiratory gated treatment is typically determined by the position of an external marker block relative to a preset gating window (15, 28). Such gating in principle assumes a constant linear relationship between external and internal motion and corresponds to our simplest scenario of ECM 1 with no model updates during treatment (Strategy A). However, due to hysteresis motion a simple linear ECM will not always provide an accurate model fit between external and internal motion (**Figure 2**, top, **Table 1**). Additionally, primarily due to baseline drift during treatment, scenarios without ECM updates could not accurately estimate the internal motion throughout the fractions (**Figure 6A**, **Table 1**). These results confirm work by Ge et al., who concluded, from fluoroscopic imaging during respiratory gating, that “inconsistent respiratory gating accuracy occurred within individual treatment fractions” (15).

For improved ECM fit accuracy, we showed that the augmented linear model (ECM 2) including a term to handle hysteresis motion provided a good compromise between fit accuracy and robustness towards irregular motion. More complex quadratic models (ECM 3 and 4) provided better or similar model fits but were less robust towards irregular motion. For improved internal motion estimation during treatment, three different ECM update strategies (B-D) were investigated. Strategies B and C simulated continuous ECM update every 3 s (0.33 Hz), with Strategy C providing a faster and simpler update based only on the last five samples and accounting only for baseline drifts between internal and external motion. Strategy D employed on-demand ECM building by 20 s modelling followed by 60 s estimation, thereby simulating full ECM generation before delivery of individual treatment fields. With a mean RMSE of 0.41 ± 0.14 (LR), 1.02 ± 0.33 mm (CC) and 0.78 ± 0.48 mm (AP), the best result was obtained by ECM 2 combined with strategy C (**Figure 5**, **Table 1**). While the ECM position estimation during treatment cannot be expected to be better than the fit accuracy itself, the RMSE of ECM 2 combined with Strategy C throughout the fractions was only 0.04 mm (LR), 0.10 mm (CC) and 0.15 mm (AP) larger than the fit accuracy during the first 60 s (**Table 1**, **Figure 6B**). Hence, frequent adaptation to baseline drift (parameter a in ECM 2, Equation 2) ensured accurate hybrid motion monitoring, indicating that the other parameters in the ECM (b, c, τ) remained stable during the treatment sessions. While updating only the constant term with 0.33 Hz frequency (Strategy C) was the best update strategy for the linear ECMs 1 and 2, a full update of all ECM parameters tended to be a better strategy for the augmented quadratic ECM 3 (Strategy B, **Table 1**). This could indicate a tendency of over-fitting by the more complex ECM 3.

A standard linear accelerator does not offer hybrid motion monitoring as a commercial solution and is only equipped with monoscopic kV imaging. Still, the hybrid monitoring methods investigated in this study could be implemented and integrated in a clinical workflow with proper software updates. The internal 3D motion (e.g. of implanted fiducial markers) needed to establish the pre-treatment ECM can be estimated with high accuracy from marker segmentation in a sequence of 2D x-ray images acquired from different directions by the method of KIM (29). The projection images acquired during a standard CBCT scan constitute a suitable and widely available dataset for this as shown in several studies that have determined 3D marker motion in patient coordinates from CBCT projections by the KIM method (2, 3, 30–33). It provides the internal motion for typically 60 s with 11-15 Hz temporal resolution. By applying external monitoring with a standard gating system during setup CBCT acquisition, the ECM may therefore be generated without extra imaging dose or workflow procedures. As demonstrated by Cho et al. monoscopic imaging can perform just as well as stereoscopic imaging for ECM parameter estimation (33). During rotational VMAT treatments, the 3D internal 0.33 Hz motion monitoring for ECM update strategies B and C is possible by combining the KIM method with 2D triggered kV imaging, which is available on the most widespread conventional treatment platform (Varian TrueBeam (34),). This was demonstrated during liver SBRT treatments by Bertholet et al. who named their hybrid real-time monitoring method COSMIK (25). The on-demand 20 s 3D motion monitoring for update strategy D may also be implemented on a conventional linear accelerator by combining KIM with monoscopic kV fluoroscopy during a 120 degree gantry rotation as demonstrated by Keall et al. (29).

Bertholet et al. simulated hybrid tumor motion monitoring for the same patient cohort as the present study using a scenario similar to ECM 2 with update strategy C and reported RMSE very similar to ours (LR: 0.46 mm, CC: 1.12 mm, AP: 0.82 mm) (25). For comparison, they achieved similar or smaller errors by full kV monitoring with the KIM method (LR: 0.50 mm, CC: 0.13 mm, AP: 0.53). For an ECM strategy without updates, Bertholet et al. reported smaller mean RMSE than ours (CC: 1.78 mm versus ours of 2.65 mm). This difference is due to shorter motion traces applied by Bertholet et al. who used the longest unbroken motion trace while the current paper used longer motion traces that spanned sections of data missing e.g. due to loss of the Calypso signal during setup CBCT acquisition or couch rotations at some fractions.

Different hybrid motion monitoring scenarios have been investigated in previous studies however without long sequences of independently measured internal and external motion. Poels et al. compared the Cyberknife Synchrony and Vero systems that both apply hybrid kV/optical motion monitoring (21). The Cyberknife system applies ECM updates every minute by adding the new x-ray target position to the data and uses second order polynomial ECMs with a fallback strategy to a linear model if the external breathing amplitude exceeds the amplitude during ECM training. This strategy is supported by our study, where the quadratic models were less robust towards irregular motion and sometimes had large estimation errors when the external motion amplitude was much larger than during the model fitting period (see ECM 3 in **Supplementary Figure S3**). The Vero applies a second order polynomial ECM dependent on both the position and speed of the external signal and uses stereoscopic 0.5 Hz imaging during treatment for ECM validation. Both systems perform full ECM rebuilding if large errors (Cyberknife > 5mm, Vero > 3mm) are detected in stereoscopic images. This requires treatment interruption and imaging over an extended period of time. Our data indicate that a simpler strategy of only constant (baseline) term rebuilding may be advantageous because it in principle only requires one image (or a few images for better robustness) and still mitigates most of the errors. For a group of liver and lung patients, Poels at al (21). reported no significant accuracy differences between the Cyberknife and Vero ECMs (95^th^ percentile ECM error = 3.7 - 4.1 mm without model updates) over extended periods of time (average 7 min). Their study was based on sparse 0.5 Hz stereoscopic imaging of internal motion. Another recent study compared the Radixact and Cyberknife hybrid systems (23). The Radixact hybrid system is an adaptation of the Cyberknife Syncrony Respiratory Tracking System where the internal motion is estimated by monoscopic x-ray imaging. The study reported RMSE in the range of 0.2-3.5mm for the two systems, with a slight tendency for better performance by the Radixact system. However, the study only included three traces of lung tumor motion where the internal motion itself was estimated based on Cyberknife ECMs (35). It clearly stresses the need for databases containing complete long-duration motion traces with independently measured internal and external motion.

In an early study, Seppenwoolde et al. analyzed shorter high-frequency motion traces with an average length of 82 s of combined internal x-ray monitoring and external surface monitoring by a laser system (26). Consistent with the present study, hybrid monitoring (Cyberknife model) was found to be able to reduce errors to a few mm though no direct population error metric was reported. A small trend between ECM update frequency and accuracy was observed. Based on a database of Cyberknife lung and pancreas treatments, Malinowski et al. also confirmed the importance of ECM model updates to reach mean tumor estimation errors below 2 mm (36). In an initial assessment of the Vero system, Depuydt et al. analyzed seven lung (training) sessions with combined external optical and internal 1 Hz x-ray monitoring for approximately 60 s and found that the 2D ECM error was below 3.08 mm (90^th^ percentile) for that system without model updates (22).

Compared to direct internal monitoring by fluoroscopic imaging, hybrid monitoring has the advantages of reduced imaging dose, usability for non-coplanar fields where imagers cannot be deployed, increased robustness towards occasional erroneous marker segmentation and low system latency (37). Compared to standard external marker-based gating at conventional linear accelerators, ECMs could provide accurate continuous estimation of internal motion during treatment for triggering based on internal gating levels. With respect to real-time tumor tracking treatments, the reduced latency of an external optical signal compared to x-ray based monitoring is an important advantage since it reduces the need for motion prediction algorithms in tracking treatments (37–39). The continuous motion signal could also be employed for (real-time) assessment of the actual target dose delivered during treatment taking the intrafraction motion into account (40).

Limitations of the present work include the missing sections in some of the motion traces and different trace lengths, which resulted in different numbers of included fractions as function of time (**Figure 6**, **Supplementary Figure S1**). Also, only a limited number of ECMs and ECM update strategies were investigated, focusing on the previously demonstrated hybrid methodology of COSMIK (25) and similar strategies that may be realized at a conventional LINAC. Future work should in more detail investigate the performance of different available systems and possible improvements hereof including optimization of the training duration used for pre-treatment ECM building, the imaging interval between ECM update imaging, and the number of images (i.e. motion history length) used for ECM updates. It would also be advantageous to include detection of irregular external motion that may signal a hampered ECM e.g. due to coughing, deep breaths or the patient falling asleep. This could mitigate the risk of ECM building during non-representative motion periods and trigger ECM updates when needed during treatment. The present study also only considered rigid motion. While not supported by the current standard hardware and software solutions at a clinical linear accelerator, an ideal hybrid ECM-based monitoring system should include detection and modelling of deformations and rotations for optimal targeting accuracy (3, 7). Monitoring of individual internal marker motion (3) and surface-guided radiotherapy (41) may play a role in this respect. Finally, verification of the model choice and update strategy in an independent dataset of internal and external motion is warranted to confirm our findings.

In conclusion, we utilized a unique dataset of synchronized external and internal motion to investigate the accuracy of four different ECMs and ECM update strategies for hybrid motion monitoring during radiotherapy. Of the investigated scenarios, an augmented linear ECM with continuous frequent update of the baseline term in the ECM model provided the best compromise between fit accuracy and robustness towards irregular motion.

## Data Availability

The data used in this study will be shared upon reasonable request.
