# Supplementary material for: Accuracy of four models and update strategies to estimate liver tumor motion from external respiratory motion
Source: Front Oncol. 2024 Sep 24;14:1470650. doi: 10.3389/fonc.2024.1470650 (PMC11458717; doi:10.3389/fonc.2024.1470650)
Supplement: Supplementary file 1 [file DataSheet1.pdf]

Supplementary Figures

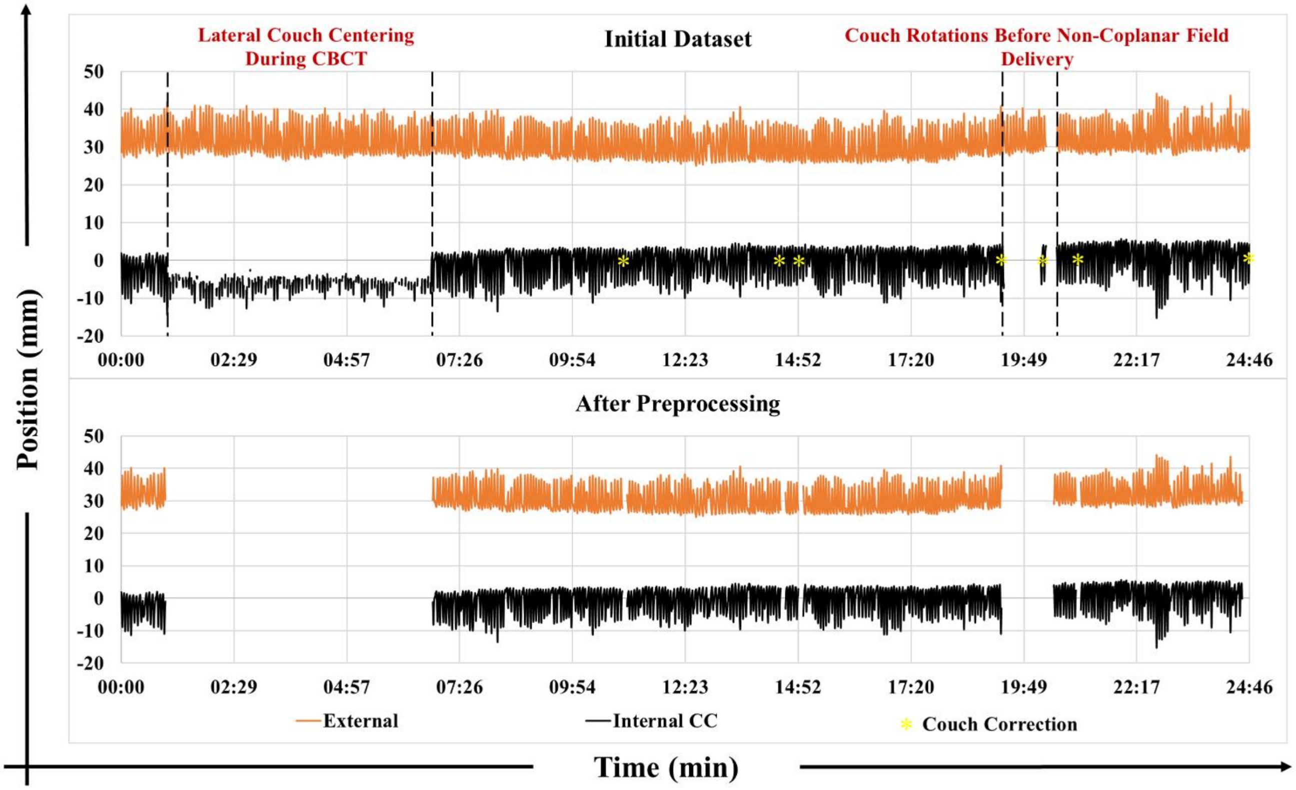

Supplementary Figure 1. An example of external marker block motion (orange) and internal cranio-caudal tumor motion (black) traces before (top) and after (bottom) removal of sections with missing or unreliable data due to the lateral couch centering during CBCT, couch rotation before non-coplanar field delivery, and couch corrections (marked with yellow *).

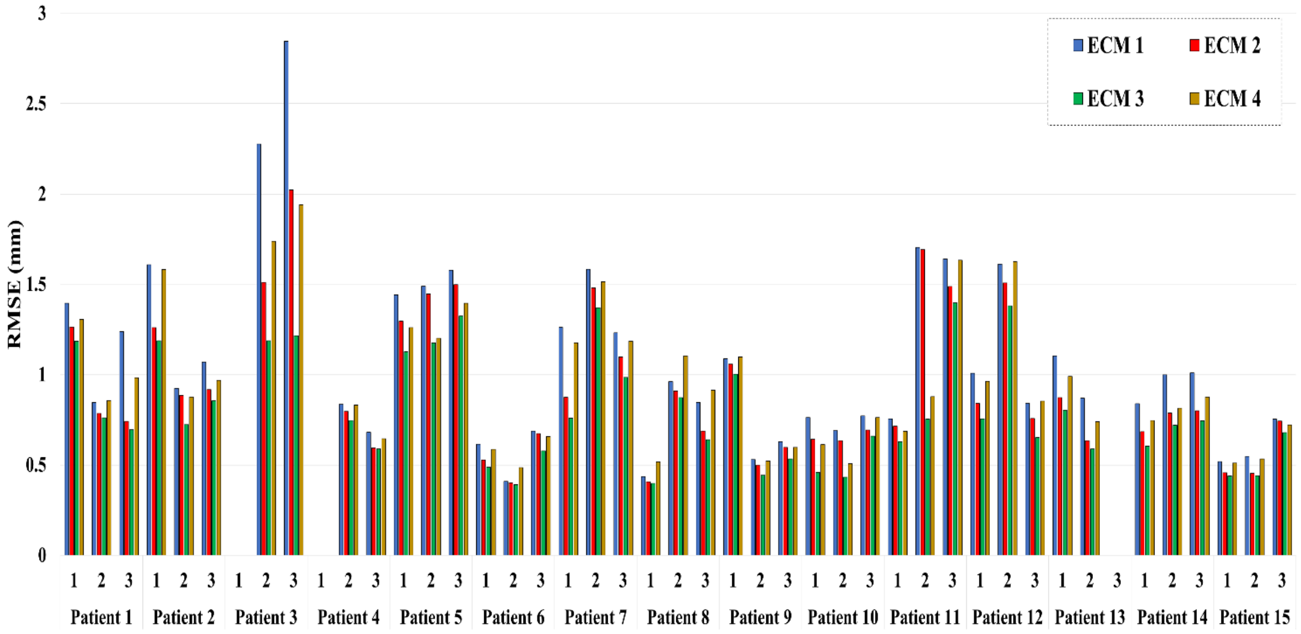

Supplementary Figure 2. Fit accuracy in the cranio-caudal direction for the four external-internal motion correlation models (ECM) for each patient and fraction.

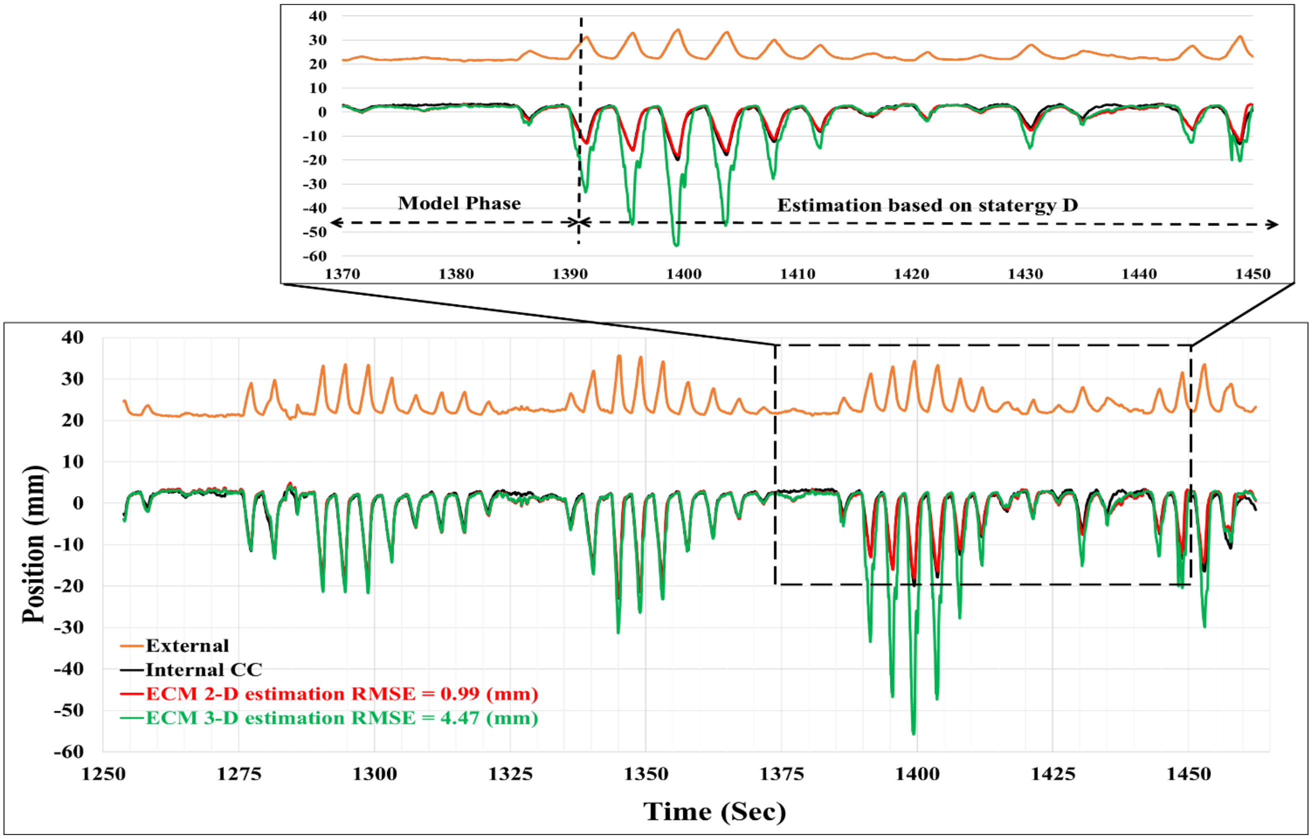

Supplementary Figure 3. Example comparing ECM 2 (augmented linear model) and ECM 3 (augmented quadratic model) for update strategy D (on demand ECM generation by 20 s monitoring). The curves show external anterior-posterior motion (orange), internal cranio-caudal motion (black) and estimated internal motion by ECM 2 (red) and ECM 3 (green).
